# Temporal Changes of the Oral and Fecal Microbiota after Mild Traumatic Brain Injury in Rats by 16S rRNA Sequencing

**DOI:** 10.3390/microorganisms11061452

**Published:** 2023-05-31

**Authors:** Shujuan Wang, Yanjie Shang, Zhiyun Pi, Ziqi Zhou, Xiangyan Zhang, Lipin Ren, Weibo Liang, Yadong Guo, Jifeng Cai, Changquan Zhang

**Affiliations:** 1 Department of Forensic Science, School of Basic Medical Sciences, Central South University, Changsha 410013, China; sjw2627@163.com (S.W.); shangyj@csu.edu.cn (Y.S.); cynthia090721@126.com (Z.P.); zhouwocao1101@163.com (Z.Z.); zxy196@csu.edu.cn (X.Z.); renlp87@126.com (L.R.); gdy82@126.com (Y.G.); 2 Department of Forensic Genetics, West China School of Basic Medical Sciences & Forensic Medicine, Sichuan University, Chengdu 610041, China; liangweibo@scu.edu.cn; 3 Shanghai Key Lab of Forensic Medicine, Key Lab of Forensic Science, Ministry of Justice, China (Academy of Forensic Science), Shanghai 200063, China; 4 School of Xiangya Medical College, Central South University, Changsha 410078, China

**Keywords:** mild traumatic brain injury, microbiota, brain-gut-microbiota axis, forensic identification, 16S rRNA

## Abstract

A mild traumatic brain injury (mTBI) can increase the risk of neurodegenerative-related disease, and serious long-term outcomes are often overlooked. In forensic science, the accurate identification of mTBIs can directly affect the application of evidence in practice cases. Recent research has revealed that the oral cavity and fecal microbiota play a fundamental role in deeply interconnecting the gut and brain injury. Therefore, we investigated the relationship between the temporal changes of the oral cavity and fecal bacterial communities with damage identification and post-injury time estimation after mTBI. In this study, we analyzed the oral cavity and fecal bacterial communities in mTBI rats under 12 different post-injury times (sham, 0 h, 2 h, 6 h, 12 h, 24 h, 2 d, 3 d, 5 d, 7 d, 10 d, and 14 d post-injury) using 16S rRNA sequencing technology. The sequence results revealed bacteria belonging to 36 phyla, 82 classes, 211 orders, 360 families, 751 genera, and 1398 species. Compared to the sham group, the relative abundance of the bacterial communities varied markedly in the post-injury groups. Importantly, our data demonstrated that Fusobacteria, Prevotellaceae, Ruminococcaceae, and Lactobacillaceae might be the potential candidates for mTBI identification, and 2 h post-injury was a critical time point to explore the temporal changes of mTBI injury-time estimation. The results also provide new ideas for mTBI treatment in the clinic.

## 1. Introduction

Traumatic Brain Injury (TBI) is the most common cause of death and disability, representing a serious healthcare problem [1]. Generally, TBI is a complex and common disorder resulting in different brain functional outcomes, characterized by high morbidity and mortality [2]. Currently, the clinical severity of TBI has long been classified based on the post-resuscitation Glasgow Coma Scale Score (GCS) into mild (GCS: 14–15), moderate (GCS: 9–13), and severe (GCS: 3–8) [3,4]. Mild traumatic brain injury (mTBI) was considered a benign and self-limiting injury under the subconscious, which accounts for 70% to 90% of TBI [5]. However, it has been documented that mTBIs could cause severe consequences lasting more than one year, and the serious long-term outcomes were often overlooked [6,7], such as cognitive impairment, cognitive deficits, and memory-related and information processing problems [8,9,10,11]. Notably, the long-term effects of mTBIs not only impose a huge burden on society and families but also make it a major public healthcare problem. Therefore, accurately identifying mTBIs is urgent to diagnose and prevent long-term outcomes.

In forensic science, the accurate identification of mTBIs will directly affect the application of evidence. Nevertheless, the identification of mTBIs was mostly based on subjective description and presumptive in the current practice of forensic science. Due to the lack of objective examination standards, mTBI-related accurate identification has always been difficult and focused in practice. Currently, imaging methods have been utilized to identify mTBI, such as head Computed Tomography (CT) [12], Magnetic Resonance Imaging (MRI) [5], and Magnetoencephalography (MEG) [11]. However, the imaging examinations were limited by the low sensitivity and expensive examination equipment [13], in which the accuracy of mTBI identification and post-injury time estimation was affected in practice. Therefore, despite the continuous updating of detection technology, the identification accuracy of mTBI has not been improved, and there are no objective and reliable methods to estimate the time of injury accurately. To overcome the disadvantages of existing detection methods, the potential of the microbiota as a tool to identify mTBI has been revealed.

The collection of microbial genomes is composed of bacteria, bacteriophage, fungi, protozoa, and viruses from an ecological community inside and on the surface of the human body [14]. In recent years, some studies have revealed that the alterations in the composition of the microbiota played a fundamental role in deeply interconnecting gut and brain injury prevention and recovery, which is widely accepted by the “brain-gut-microbiota axis (BGMA)” [15]. Specifically, Singh et al. [16] observed a significant reduction in species diversity after stroke, which might be a key feature of microbiota dysregulation. In Alzheimer’s disease (AD), Sun et al. [17] discovered that the gut microbial diversity had a variation decrease tendency, which included the Firmicutes and an increase in Bacteroidetes. Similarly, Nicholson et al. [18] indicated that moderate traumatic brain injury altered the gastrointestinal microbiota in a time-dependent manner, with the lower the Firmicutes level, the higher the Proteobacteria level. Among these, Dong et al. [19] demonstrated the succession of the oral microbial community as a forensic tool for postmortem interval (PMI) estimation. Thereafter, Liu et al. [20] established an accurate PMI estimation model by collecting microbial sequencing data sets of visceral organs in murines during the 15 d decomposition. Although studies have documented the role of the oral cavity and fecal microbial succession in actual cases, the microbiota could be a potential tool for PMI estimation in forensic investigation and diagnostic targets in disorders. Meanwhile, due to the microbe and brain interaction, the microbial changes might serve as a new marker for accurate mTBI identification and post-injury time estimation.

In this study, we aimed to explore the temporal changes of the oral cavity and fecal microbiota in the mTBI model and disclose the relationship between microbial community variation trends and mTBI identification by high-throughput sequencing. Notably, the results demonstrated that the variation of microbiota was related significantly to the mTBI identification and post-injury time estimation at the different classification levels.

## 2. Materials and Methods

### 2.1. Animals

The studies were approved by the Institutional Review Board of the School of Basic Medical Science, Central South University (approval code: KT2021-09). The methods were carried out by the approved guidelines, and the study was reported in accordance with ARRIVE guidelines.

Adult male Sprague-Dawley rats weighing 250–300 g were used in this study, provided by the animal center of the third Xiangya Hospital Central South University (Changsha, China). Briefly, rats were housed in a 12 h:12 h light:dark reversed light cycle at a constant temperature (24 ± 2 °C) and humidity (55 ± 5%). Standard food and water were also provided ad libitum. The rats were housed for a week before the operation to acclimatize to the laboratory environment. A total of 36 rats were randomly divided into 12 groups (*n* = 3): sham, 0 h post-injury, 2 h post-injury, 6 h post-injury, 12 h post-injury, 24 h post-injury, 2 d post-injury, 3 d post-injury, 5 d post-injury, 7 d post-injury, 10 d post-injury, and 14 d post-injury. The principle of minimizing the number of animals used and alleviating their suffering has been adhered to during the experiment.

### 2.2. mTBI Model Established

mTBI was constructed from the Feeney weight-drop contusion model, with minor modifications to the previously established model. Briefly, rats were anesthetized with sodium pentobarbital by intraperitoneal injection (40 mg/kg, i.p.), and hair was removed from the head with an electric razor and mounted on the stereotaxic frame. During the experiment, body temperature was maintained at 37 °C using a heating pad. Under sterile conditions, a 3 cm midline incision was made on the scalp, and the soft tissue was carefully exposed to the skull. Subsequently, a 5 mm craniotomy was performed using a portable drill on the center of the left parietal bone; then, the bone flap was removed to expose the intact dura. The center of the bone window was located 3 mm posterior to the bregma and 3 mm lateral to the sagittal line. Then, a weight-drop device was placed over the dura, and an impact sensor (4 mm in diameter) was set to stop at a depth of 1 mm below the diameter. After, a weight of 20 g was dropped from 24 cm above the dura through the guide tube. Whereafter, the incision was sutured intermittently and disinfected. After the operation, the rats were returned to the cage for recovery. The sham rats were subjected to the same experimental procedure without cortical impact. Animals with cerebral hemorrhage, skull fractures, or contusions were excluded.

### 2.3. Neurological Function Evaluation

To evaluate the results of neural function, an 18-point Modified Neurological Severity Score (mNSS) scale was used [21]. A detailed description of mNSS is shown in Table S1. Briefly, the mNSS is a combination of sensory (touch, vision, and proprioception), motor (abnormal movement and muscle state), and reflex tests. The evaluation was performed on all rats, and the post-injury groups were tested 2 h after awakening. All measurements were taken without the observers’ knowledge.

### 2.4. Histopathological Assessment

When exploring acute injury, subacute injury, and chronic injury, the injury time of SD rats can be set at 24 h, 7 d, and 14 d, respectively. The survival time of the 12 groups of rats depended on the group assignment. At that time, rats were deeply anesthetized (100 mg/kg sodium pentobarbital) and infused with phosphate-buffered saline (PBS) through the heart, followed by 4% paraformaldehyde (PFA). The whole brain was removed from the skull immediately and elaborately, then fixed with 4% PFA for 4 d. Subsequently, the paraffin-embedded brain tissue was used for histopathological examination. The successive coronal sections (5 µm) of injured brain tissue were stained with Hematoxylin and Eosin (H &E) to determine the extent of the damage.

### 2.5. Samples Collection

Samples of bacterial communities were collected in the sham group (0 h) and 0 h, 2 h, 6 h, 12 h, 24 h, 2 d, 3 d, 5 d, 7 d, 10 d, and 14 d post-injury groups. The oral cavity was softly swabbed with a sterile cotton swab soaked with sterile water for 15 seconds, and the uncontaminated feces were collected into sterile tubes. Then, the cotton applicator was placed into a 1.5 mL sterile Eppendorf tube with 1 mL sterile ultrapure water. The oral cavity and feces samples were frozen at −80 °C for later use.

### 2.6. DNA Extraction

Total bacterial DNA was extracted from the SD rats’ oral cavity and fecal samples using a DNA Extraction kit following the manufacturer’s instructions. The concentration and purity of DNA were verified by NanoDrop 2000 and 2% agarose gel electrophoresis. The genome DNA was used as the template for PCR amplification with the barcoded primers and TKs Gflex DNA polymerase (Takara, OE Biotech Co., Ltd., Shanghai, China) to ensure amplification efficiency and accuracy.

### 2.7. PCR Amplification

The V3-V4 hypervariable regions (forward primer: 5′-TACGGRAGGCAGCAG-3′, reverse primer: 5′-AGGGTATCTAATCCT-3′) 343F/798R primer was used to conduct the PCR reactions. The PCR reaction consisted of 15 µL Gflex PCR buffer, 1 µL forward and reverse primers, 0.6 µL of TKs Gflex DNA polymerase, and 50 ng of template DNA in a 30 µL reaction volume. The PCR reactions were as follows: 94 °C for 5 min, 26 cycles of 94 °C for 30 s, 56 °C for 30 s, and 72 °C for 20 s. Finally, it was extended for 5 min at 72 °C.

### 2.8. 16S rRNA Sequencing

Trimmomatic software (version 0.35) [22] was used to preprocess paired-end reads and detect and cut off fuzzy bases (N). Specifically, the low-quality sequences with an average quality score below 20 were cut off using the sliding window trimming approach. After trimming, paired-end reads were assembled using FLASH (version 1.2.11) [23]. The assembly parameters were: 10 bp of minimal overlapping, 200 bp of maximum overlapping, and 20% of maximum mismatch rate. Sequences were further denoised as follows: reads with ambiguous, homologous sequences or below 200 bp were abandoned. Reads with 75% of bases above Q20 were retained, and reads with chimera were detected and removed by QIIME software (version 1.8.0) [24].

### 2.9. Sequencing Data Analysis

The Search software (version 2.4.2) [25] was adopted to cluster the sequences, and a threshold of 97% similarity was used to assign similar sequences to operational taxonomic units (OTUs). The representative read of each OTU was selected using QIIME (version 1.8.0) package. In addition, the representative reads were annotated and blasted against the Silva database (Version 138) using the RDP classifier (confidence threshold was 70%) [26]. The Unite database (ITSs rDNA) was annotated and blasted against using BLAST [27].

Alpha diversity analysis was utilized to assess species richness and distribution evenness of samples by calculating different diversity indexes, including the Chao1 index, Shannon index, and Simpson index. Based on the diversity index, the different significance of the diversity index in different groups can be calculated by alpha diversity and Kruskal–Wallis/Wilcoxon algorithms. Beta diversity analysis was an assessment of community similarities and differences among different groups. The comparison of differences was mainly based on OTU sequence similarity or species classification and distribution, including principal components analysis (PCoA), non-metric multidimensional scaling (NMDS), and unweighted pair group method with arithmetic mean (UPGMA).

### 2.10. Statistics Analysis

One-way analysis of GraphPad Prism 8 was used for the neurological function evaluation in the sham group and post-injury groups. The values indicated the higher score of mNSS with more severe injury and the lower score of mNSS with milder injury. Two-way ANOVA was also used for the samples of α-diversity in rats sustaining mTBI over all time points as measured by the Chao 1 index, Shannon index, and Observed Species to detect the degree of community richness and to evaluate diversity in the microbial community using Origin 2021.

## 3. Results

### 3.1. Well-Established mTBI Model

**Figure 1:**
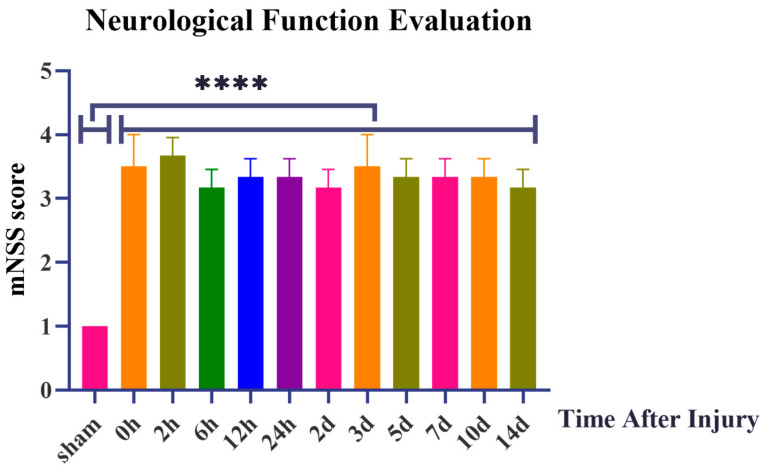
Neurological Function Evaluation by 18-score modified Neurological Severity Score points (mNSS). ****: *p* < 0.0001.

In this study, a total of 36 rats participated in the experiment of the mTBI model construction, and no death or massive bleeding occurred in rats during the whole operation. All rats regained consciousness 30–45 min after intermittent scalp sutures. As to their behavior, most rats showed indifference, poor appetite, and weak panic reflex after awakening. In the sham group, scores of the movement test, balance beam test, and other reflexes were normal. Virtually, there was a statistical difference between the sham group and the post-injury groups in the assessment of neurological function by 18-score mNSS, as shown in Figure 1. The values in Table S1 indicated the higher score of mNSS with more severe injury and the lower score of mNSS with milder injury. In the sham group, it can be speculated that the 1 score may be caused by the scalp injury rather than the appearance of divine function impairment. In addition, minor signs of injury after surgery were understandable and within the normal range. In the post-injury groups, the score ranged from 3 to 4.5 and maintained a steady trend. It can be seen that it met the standard of assessment of mild injury score from 1 to 6 points in the 18-score mNSS; the higher score of mNSS with more severe injury and the lower score of mNSS with milder injury. In the sham group, the score was 1. In the post-injury groups, the score ranged from 3 to 4.5 and maintained a steady trend.

**Figure 2:**
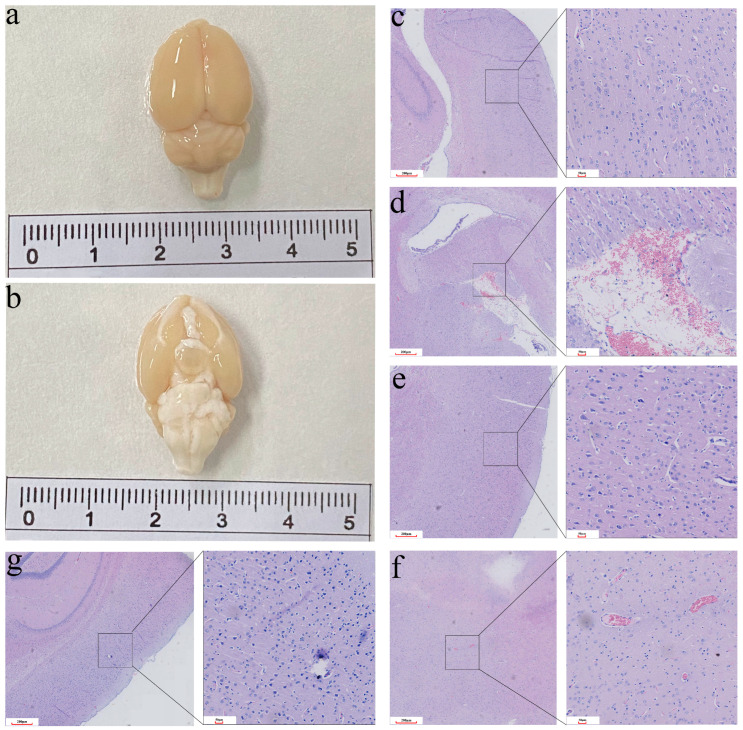
The changes in morphology and histology in the mTBI rat models. (**a**,**b**) The complete brain with no obvious cerebral contusion. (**c**) Control group. (**d**) Superficial cortical injury. (**e**) Slight edema. (**f**) Histopathologic changes of congestion. (**g**) Infiltration of inflammatory cells and neuronal swelling.

After deep anesthesia in the post-injury group, the whole brain was extracted from the skull without defect and obvious cerebral contusion (Figure 2a,b). Compared with the sham group, the neuropathological pathology of all the post-injury groups was significantly different. Specifically, neuropathological pathology showed superficial cortical injury, slight edema, and signs of hypoxia in the brain tissue (Figure 2c–e). However, there was no obvious cerebral parenchymal hemorrhage. According to the H&E staining, histopathologic changes of congestion, infiltration of inflammatory cells, and neuronal swelling were observed in the cerebral cortex (Figure 2f,g). In the present study, the morphological and microscopic histopathological observation of the brain revealed the same degree of damage in the post-injury groups. In summary, it indicated that a stable and repeatable rat mTBI model had been constructed, which can be used in subsequent experiments.

### 3.2. Summary of Sequencing Results

**Figure 3:**
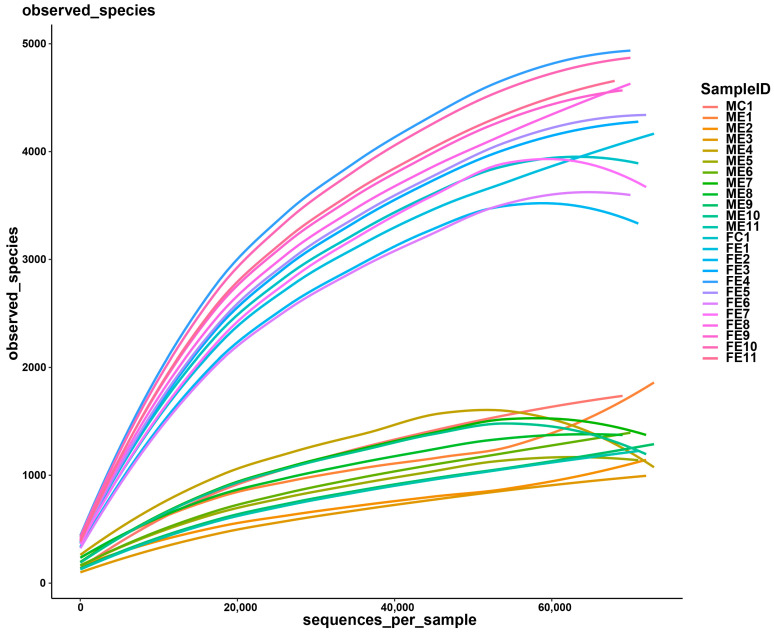
Rarefaction curves of observed species number clustered at 97% sequence identity across the oral cavity and fecal samples.

Thirty-six oral cavity samples and thirty-six fecal samples were collected in 12 groups (sham, 0 h, 2 h, 6 h, 12 h, 24 h, 2 d, 3 d, 5 d, 7 d, 10 d, and 14 d post-injury). All samples were sequenced successfully. After quality filtering and removing a low number of sequences, the clean tags ranged from 71,151 to 78,591, among which the valid tags retained by removing chimerism ranged from 59,683 to 73,562. For each sample, the average length ranged from 411.89 bp to 423.44 bp. The number of OTU ranged from 536 to 5600 at a 97% identity threshold. According to the rarefaction curves (Figure 3), the species representation in each sample had approached the plateau.

### 3.3. Bacterial Community Composition Analysis

The sequence results revealed bacteria belonging to 36 phyla, 82 classes, 211 orders, 360 families, 751 genera, and 1398 species. Detailed characteristics of each sample are listed in Table 1.

**Figure 4:**
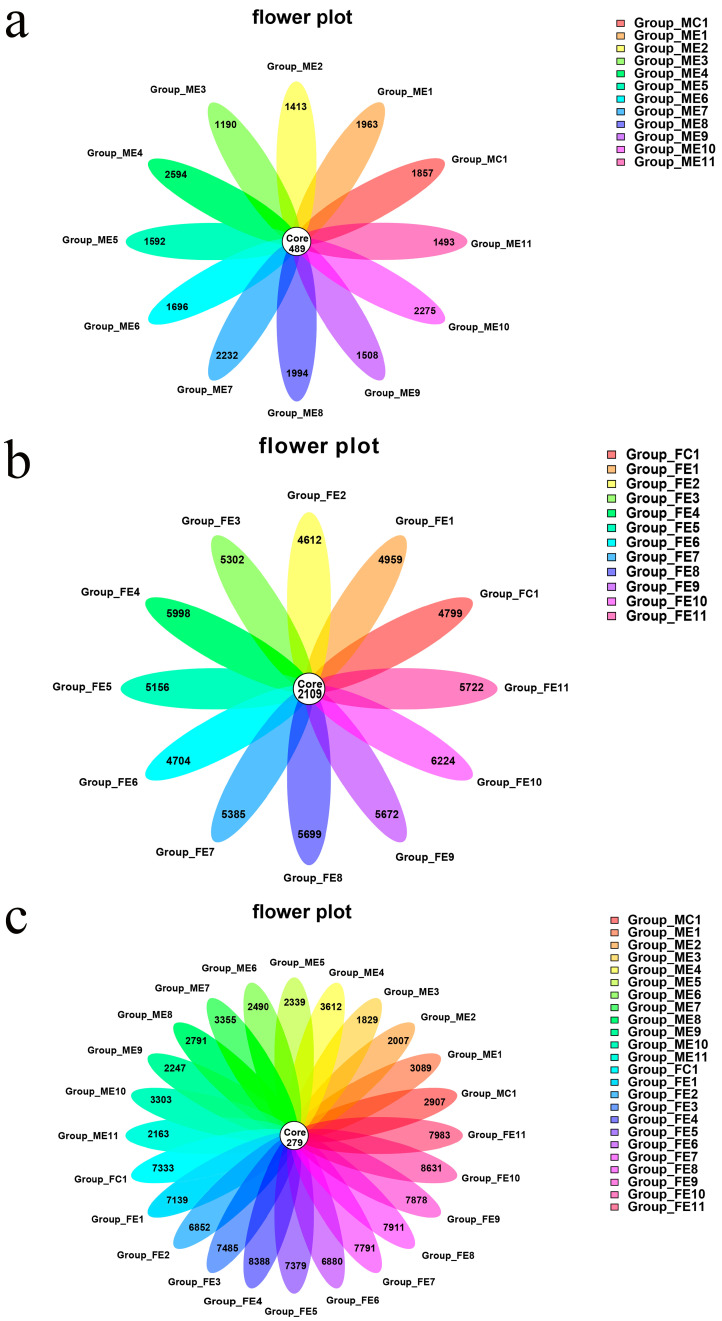
Shared Operational Taxonomic Unit (OTU) analysis of the different communities. Flower diagram showing the unique and shared OTUs in the different post-injury groups: (**a**) for the 12 oral cavity groups communities, (**b**) for the 12 fecal groups communities, and (**c**) for the 12 oral cavity groups and 12 fecal groups communities. Sample names refer to samples as described in Table 1.

A flower diagram was utilized to compare the similarities and differences between the communities in the different groups (Figure 4). The 12 oral cavity group communities had 489 OTUs in common (Figure 4a). The 12 fecal sample group communities had 2109 OTUs in common (Figure 4b). The oral cavity groups and 12 fecal sample groups communities had 279 OTUs in common (Figure 4c). According to the flower diagram, the number of OTUs in fecal samples was more significant than that in oral cavity samples. More importantly, this study proved that the fecal microbiota was more abundant than the oral cavity.

### 3.4. Bacterial Community Diversity Analysis

**Figure 5:**
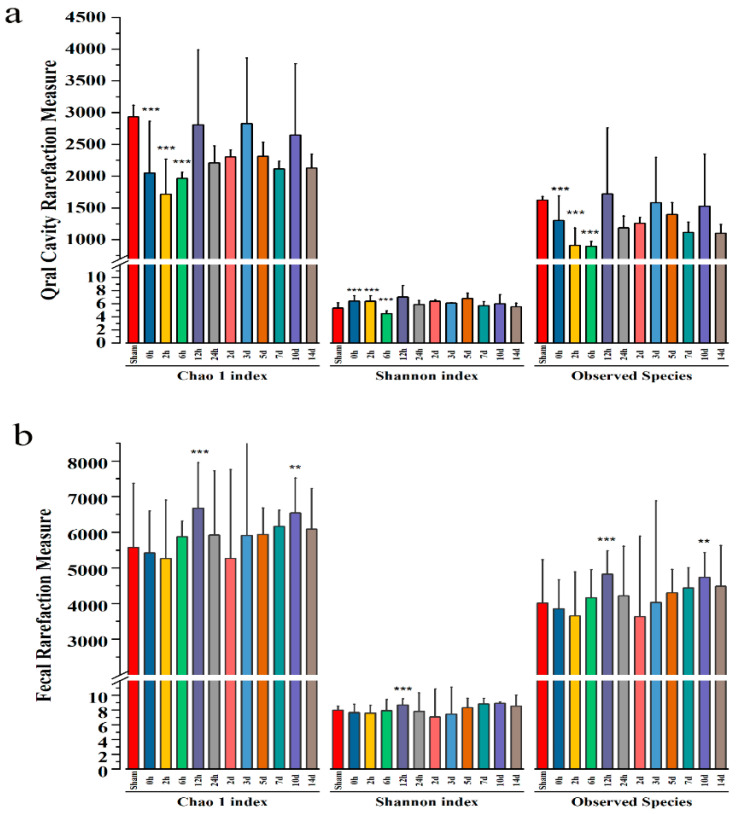
(**a**) The oral cavity indices of α-diversity in rats sustaining mTBI over all time points as measured by the Chao 1 index, Shannon index, and Observed Species. The Chao 1 indices and Observed Species indices demonstrated a trend toward a lower number of species by 2 h post-injury. (**b**) The fecal indices of α-diversity in rats sustaining mTBI over all time points as measured by Chao 1 index, Shannon index, and Observed Species. The Chao 1 indices and Observed Species indices demonstrated a trend toward a lower number of species by 2 h post-injury and 2 d post-injury. ***: *p* < 0.001, **: *p* < 0.01.

According to the alpha-diversity, the estimator of Chao 1 indices and Observed Species indices reflected the degree of community richness. In contrast, the Shannon indices were used to evaluate diversity in the microbial community (Figure 5). In the oral cavity rarefaction measure, the Chao 1 and Observed Species indices demonstrated a trend toward a lower number of species by 2 h post-injury, and this assessment of α-diversity reached statistical significance. Additionally, there was a decrease in the Shannon indices of the 6 h post-injury, indicating that the diversity of oral cavity microbiota reduced after mTBI (Figure 5a). In the fecal rarefaction measure, the Chao 1 and Observed Species indices demonstrated an increasing trend of species number at 12 h and 10d after injury. Meanwhile, the Shannon index increased 12 h after injury, indicating that fecal microbial diversity increased after mTBI (Figure 5b).

**Figure 6:**
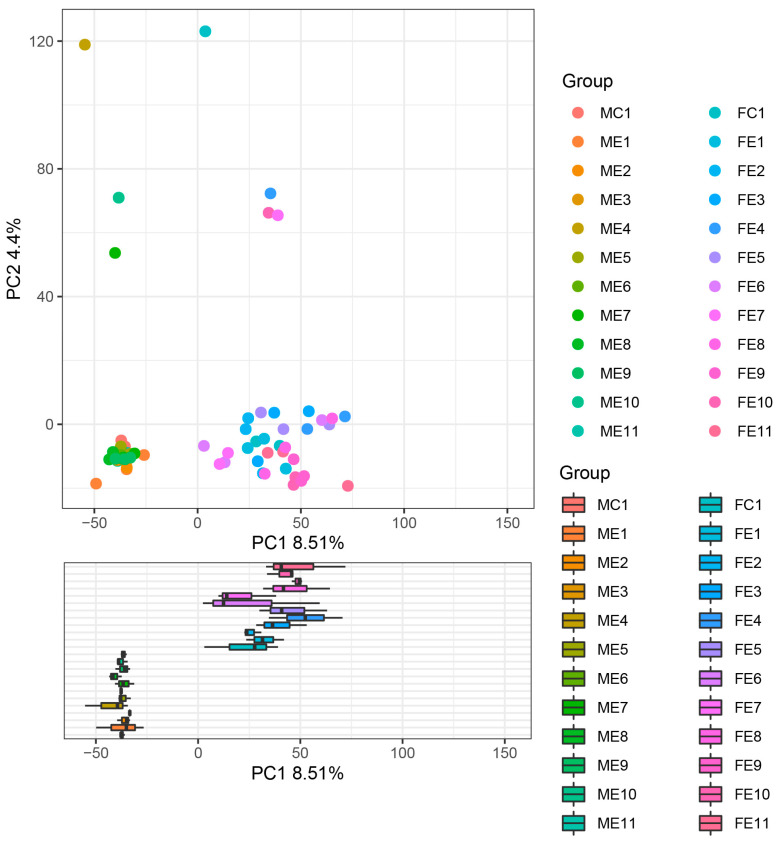
Two-dimensional subsequent distance analysis Principal Components Analysis (PCA) of the oral cavity and fecal samples. The point on the plot represents the oral cavity and fecal microbial composition of an individual rat, with orange points and blue points representing the sham group. Sample names refer to samples as described in Table 1.

According to beta diversity, the Principal Components Analysis (PCA) of the oral cavity and fecal samples cluster provided the mTBI injury in a two-dimensional pattern for subsequent distance analysis, as shown in Figure 6. The point on the plot in Figure 6 represented the oral cavity and fecal microbial composition of an individual rat, with orange points and blue points representing the sham group. The variance in the microbial composition of the oral cavity and fecal microbial communities was evident. Principal Components 1 (PC 1) and Principal Composition 2 (PC 2) analysis (explaining 8.51% and 4.4% of the variance, respectively) indicated that the oral cavity and fecal microbial communities were separated over time following mTBI. Moreover, the oral cavity and fecal microbial communities were distinguished over time following mTBI, suggesting that the sequencing data were reliable.

### 3.5. Temporal Changes of Oral Cavity Microbiota

**Figure 7:**
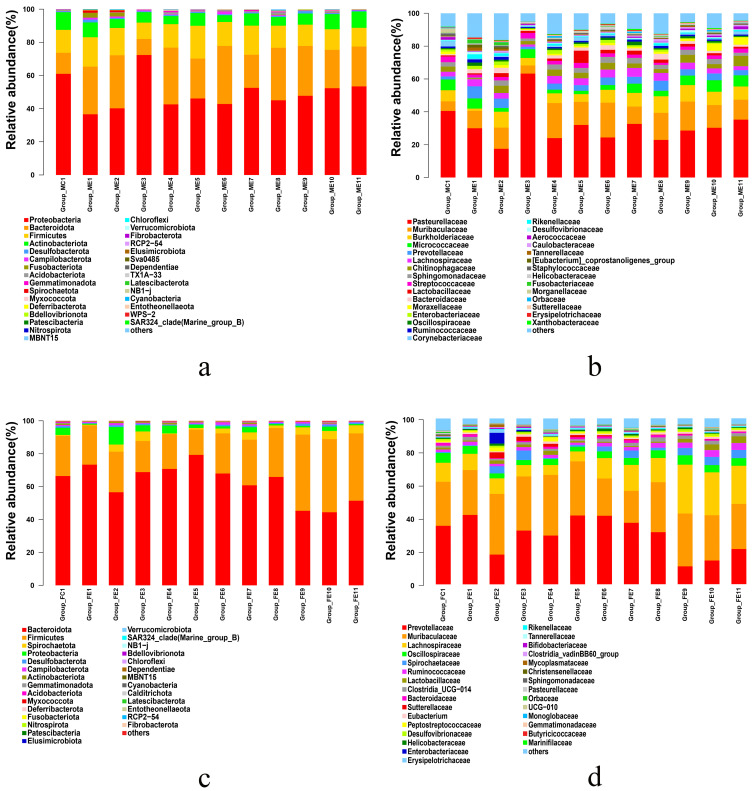
The variation of microbiota community composition in the oral cavity and fecal sample at the phylum level and the family level: (**a**) relative abundance of bacterial phyla in the oral cavity, (**b**) relative abundance of microbiota family in the oral cavity. (**c**) relative abundance of microbiota phyla in the fecal sample. (**d**) relative abundance of microbiota phyla in the fecal sample.

The results of sequences were classified at the phylum and the family levels to explore the temporal changes of microbiota community composition after mTBI. At the time of injury, the relative abundance of different microbiota communities changed obviously (Figure 7).

In the oral cavity samples (Figure 7a), the representative and most abundant phyla included Proteobacteria, Bacteroidota, Firmicutes, and Actinobacteria, irrespective of the time after injury. Interestingly, there was an apparent difference between the oral cavity samples of the post-injury and sham groups. In the sham group, the dominant phyla were Proteobacteria (61.01%) and Bacteroidota (12.67%), which comprised 73.68% of the bacteria. However, the total levels of Proteobacteria decreased throughout the time of injury by about 20% and displayed a dramatic tendency to increase in the 6 h post-injury group. The Bacteroidota, with an upward trend, were present throughout the time of injury but not in the 6 h post-injury group. Briefly, the Proteobacteria and the Bacteroidota have a large distinct tendency difference between the sham group and the post-injury groups. It may be that mTBI impacted the Proteobacteria and Bacteroidota as the time of injury increased.

At the family level in the oral cavity (Figure 7b), the representative and most abundant family was the Pasteurellaceae (31.01% ± 11.88%) and Muribaculaceae (14.14% ± 4.85%). In the sham group, Pasteurellaceae was the dominant taxon (40.52%). Specifically, from the 0 h post-injury group to the 14 d post-injury group, the Pasteurellaceae displayed a downward trend, except for the 6 h post-injury group. Interestingly, at the 6 h post-injury, the number of Pasteurellaceae increased gradually by about 23% compared to the sham group. Additionally, in the post-injury groups, the Pasteurellaceae remained relatively unchanged over time, except for a large increase in the family in the 6 h post-injury group. These data indicated that the oral cavity microbial community changed significantly at 6 h post-injury point after mTBI, while the changing trend remained stable in other post-injury groups.

### 3.6. Temporal Changes of Fecal Microbiota

In the fecal samples (Figure 7c), the Bacteroidota, Firmicutes, Spirochaetota, and Proteobacteria were the dominant phyla. However, they have different changes as the time of injury increases. Compared with the sham group, there was a significant decrease in relative abundance at the phyla level seen in Bacteroidota from 2 h post-injury to 24 h post-injury, then decreased to a stable level. Conversely, an increased relative abundance in the phyla Firmicutes was observed from 2 d post-injury to 14 d post-injury following mTBI. The Firmicutes tended to constantly increase and become the most abundant phylum from 2 d post-injury to 14 d post-injury. Additionally, the Proteobacteria tended to increase initially and then decrease. In the 2 h post-injury group, the Proteobacteria dramatically increased and took over from the Spirochaetota as the third most dominant phylum (46.72%). The above results indicated that mTBI had a more pronounced influence on the Bacteroidota, Firmicutes, and Proteobacteria.

At the family level in the fecal sample (Figure 7d), the representative and most relatively abundant included Prevotellaceae, Muribaculaceae, Lachnospiraceae, and Oscillospiraceae, irrespective of the time after injury. Specifically, the Prevotellaceae displayed an increasing tendency initially and then a decrease in the post-injury group, in which the Muribaculaceae was the predominant family. Additionally, the Ruminococcaceae dramatically changed in the post-injury groups compared to the sham group. In the post-injury groups, the Ruminococcaceae tended to constantly increase following mTBI and remained stable from 5 d post-injury to 14 d post-injury. More interestingly, Bifidobacteriaceae levels were significantly different after mTBI. Within the family Bifidobacteriaceae, it tended to increase initially and then decrease, almost vanishing from 5 d post-injury (0.083%) to 14 d post-injury (0.011%). These findings demonstrated that the probiotics and pathogenic bacteria in the fecal microbiota are markedly affected by mTBI.

## 4. Discussion

A mTBI can cause severe consequences, and the serious long-term outcomes are often overlooked. Due to the lack of objective examination standards, mTBI-related accurate identification has always been difficult and a focus of the current practice of forensic science. However, the dilemma improves when the oral cavity and fecal microbiota are applied to mTBI identification and post-injury time estimation. Based on the interaction between the microbiota and the brain, the microbial communities’ changes might serve as a new marker for mTBI identification and post-injury time estimation. Therefore, the temporal changes of the oral cavity and fecal microbiota in mTBI rat models were explored to speculate on their potential forensic applications in this study. Furthermore, considering whether these results apply to mTBI human subjects, the credibility of using oral cavity and fecal microbiota to contribute to the mTBI identification and post-injury time estimation was analyzed to supplement the study on mTBI.

### 4.1. Establishment of a Stable and Repeatable mTBI Model

This study investigated some typical characteristics and microbial temporal changes of mTBI rats by Feeney weight-drop contusion with minor modification. Based on the 18-score mNSS and histopathological assessments, the rat mTBI model has been well-established. The difference between the models is that either a craniotomy or an indirect weight drop to strike the skull was performed. Our modified model was more consistent with the manifestation of an mTBI head injury with the combination of skull fracture and cortical contusion.

### 4.2. Potential Implications for the Central Nervous System by Microbial Changes in the Oral Cavity and Feces

As a complex brain disorder, mTBI has been found to have a series of acute symptoms. Meanwhile, brain disorders are reported to be an important factor in gastrointestinal dysfunction, and the gut may even be a pathway for the spread of pathology to the Central Nervous System (CNS) [28]. The notion is supported by neurologic diseases: the effect of the gut microbiota on ischemic stroke is unique, with the most known risk factor being the reduction of SCFA-producing bacteria over time, which then affects microglia development and function [29]. Thereafter, Intracerebral Hemorrhage (ICH) has been reported to be associated with dysbiosis of the gut microbes. The neurological function after ICH can be improved by fecal transplant, although the exact bacteria population responsible for the effect remains uncertain [30]. Notably, there is growing evidence of the functional influence on the bi-directional communication between the gut and brain. In addition, the oral cavity microbiota also plays an essential role in the human microbial community and health [31]. Specifically, the synergistic action and interaction of various oral microorganisms could help the body against the invasion of external stimulation. At the same time, dysbiosis of the microbiota could contribute to oral and systemic diseases [32]. In recent years, studies have shown that ectopic colonization of oral cavity microorganisms in the healthy gut may contribute to the physiological development and maintenance of gut immunity. Similarly, specific oral bacteria and ectopic colonization under certain conditions might be associated with the pathogenesis of gastrointestinal disease [33]. In this study, we selected the oral cavity and feces as the microbial sampling points to explore the changes in microorganisms after mTBI, which proved to be scientific and objective for subsequent analysis.

### 4.3. Temporal Phyla-Level Changes of Fusobacteria in the Oral Cavity

The oral cavity is the main gateway to the human body, harboring 770 species of bacteria, which has the second-largest and most diverse microbiota after the gut. Within the human oral cavity microbiota, the primary members of the phyla were Firmicutes, Fusobacteria, Proteobacteria, and Actinobacteria [34]. However, in the oral cavity of the mTBI model, the dominant members of the phyla were Proteobacteria, Bacteroidota, Firmicutes, and Actinobacteria. The alternation of Fusobacteria in the mTBI post-injury groups was comparatively significant compared to the sham group. In the 0 h post-injury group and the 2 h post-injury group, the relative abundance of Fusobacteria reached 2.93% and 2.42%, respectively, but was extremely low in other post-injury groups. A recent study documented that the Fusobacteria was associated with tumors and shaped the tumor microenvironment by altering the cytotoxic function of tumor infiltration [35]. Meanwhile, previous studies revealed that 2 h post-injury was the most suitable time point to distinguish the microbial community. Therefore, the relative abundance variation of Fusobacteria in the mTBI experiment groups was reliable. In our study, we concluded that the specific temporal expression of Fusobacteria could be used as a community indicator for mTBI identification and post-injury time estimation.

### 4.4. Temporal Family-Level Changes of Prevotellaceae in the Oral Cavity

The dominant members were Pasteurellaceae, Muribaculaceae, Burkholderiaceae, and Micrococcaceae in the family level of oral cavity samples. However, the data demonstrated that Pasteurellaceae changed substantially in the 6 h post-injury group, which meant that the microorganism community of the 6 h post-injury group differed from other post-injury groups. Moreover, the current study has shown that Prevotellaceae were significantly increased in the mTBI post-injury groups compared to the sham group. Specifically, the relative abundance of Prevotellaceae increased in all the mTBI post-injury groups. Previous studies showed that Prevotellaceae could release an inflammatory substance called H2S from sulfur-containing amino acids that contribute to colonic tumor development [36]. Thereafter, Geva-Zatorsky and his colleagues demonstrated that ectopic intestinal colonization by specific oral cavity microbiota under certain conditions might be associated with the pathogenesis of the gastrointestinal tract [37]. Therefore, it could be speculated that Prevotellaceae could contribute to the in-depth study of the microbial-gut-brain axis and expect to become a new marker for mTBI identification and post-injury time reference.

### 4.5. 2 h Post-Injury Was a Critical Time to Explore the Temporal Phyla-Level Changes in Fecal Samples

The Human Microbiome Project (HMP) reported that the Firmicutes and Bacteroidetes were the dominant phyla in the human gut, followed by other enterotypes such as Prevotella, Bacteroides, and Ruminococcus. The role of gut microbiota in homeostasis and maintaining health continues to be revealed; suffice it to say that an intact microbiota is essential for gut and body health [38]. In the fecal samples of the mTBI model, the dominant members of the phyla were Bacteroidota and Firmicutes, followed by Spirochaetota and Proteobacteria. The relative abundance of Firmicutes was maintained at baseline level until 24 h post-injury. In comparison, it appeared to have an increasing tendency and became the dominant taxon from 2 d post-injury to 14 d post-injury. Thus, the 2 h post-injury was a critical time to explore the temporal changes of fecal microbiota for mTBI identification and damage time estimation, consistent with the findings of oral cavity microorganisms.

### 4.6. Potential Significance of Temporal Family-Level Changes of Microbiota in Fecal Samples for Forensic Identification

The dominant numbers at the family level are Prevotellaceae and Muribaculaceae, followed by Lachnospiraceae and Oscillospiraceae in the fecal samples. Our data demonstrated that the relative abundance of Prevotellaceae first increased in the 0 h post-injury and then decreased from 2 h post-injury to 14 d post-injury and eventually maintained a low relative abundance below the baseline. Interestingly, Ruminococcaceae, Bifidobacterium, and Lactobacillaceae showed an apparent upward tendency from 3 d post-injury to 14 d post-injury. Recently, a study reported that the abundance of Lactobacillus is higher, while Prevotella, Clostridiumcoccoides, and Bacteroides fragilis are low in fecal samples of PD patients [39]. Thereafter, low Prevotella numbers might decrease mycoprotein synthesis and increase intestinal permeability, potentially shifting the balance of microbes in the colon to a more inflammatory phenotype. Moreover, in rat models, the hydrogen sulfide secreted by Prevotella has been reported to have a protective effect on dopaminergic neurons [40]. Further studies observed that Ruminococcaceae and Bifidobacterium tend to be elevated in the fecal samples of PD patients [17,33]. The putative cellulose-degrading bacteria from Ruminococcus were significantly reduced, while the putative pathobionts from Streptococcus and Proteus were significantly increased compared to the control group. Some bacteria could produce neurotoxins such as Streptokinase and Streptomycin, which might result in permanent nerve damage [41]. Therefore, we speculated that mTBI might have similar symptoms as PD with gut microbiota imbalance and alteration of microbial metabolites. In the fecal samples of mTBI, Prevotellaceae, Ruminococcaceae, and Lactobacillaceae were the potential candidates for mTBI identification and post-injury time estimation.

## 5. Conclusions

In conclusion, we sought to explore the relationship between the oral cavity and fecal microbial variation and the identification and post-injury time estimation of mTBI through 16S rRNA sequencing technology. Moreover, the results of sequences were classified from phylum and family level to explore the construction of bacterial communities after mTBI. In this study, the data demonstrated that Fusobacteria, Prevotellaceae, Ruminococcaceae, and Lactobacillaceae might be the potential candidates for mTBI identification, and the 2 h post-injury was a critical time for exploring the temporal changes of mTBI damage time estimation. Considering the results of this study, the temporal changes in the oral cavity and fecal microbial community provided new ideas for mTBI identification and post-injury time estimation in forensic science and modification of microbiota for mTBI treatment in the clinic. Despite the profound findings, the present study still had several shortcomings. First, a pathological link between temporal changes of microbiota and mTBI was observed but still at the correlational stage. Therefore, further validation tests may assist in clarifying causality and finding markers that contribute to the diagnosis of mTBI. Second, the results obtained from rats had a certain reference value, but they could not entirely reflect the actual situation of the human body. In parallel and essential are further experiments studied to clarify the potential connection between microbiota changes and mTBI in the real situation of the human body.

## Figures and Tables

**Table 1:** Operational taxonomic unit (OTU)-based diversity index in rat oral cavity and fecal samples in the mTBI model.

Sample Name	Sample Group	Sample Region	Sample Time	Clean Tags	Valid Tags	OTUs	Chao1 Index	Shannon Index	Observed Species
MC1.1	Group MC1	oral	Sham (0 h)	75,337	67,337	1798	3116.86	4.52	1682
MC1.2				76,332	68,857	1742	2886.53	5.28	1612
MC1.3				75,656	69,597	1712	2802.71	6.17	1582
ME1.1	Group ME1	oral	0 h post-injury	71,304	66,447	546	819.09	7.23	528
ME1.2				77,807	73,239	1869	2866.49	5.47	1691
ME1.3				77,425	67,121	1760	2474.31	6.58	1685
ME2.1	Group ME2	oral	2 h post-injury	72,793	68,057	535	884.99	7.22	514
ME2.2				76,474	72,318	1166	2270.56	5.70	1042
ME2.3				75,877	71,371	1289	1987.34	6.28	1185
ME3.1	Group ME3	oral	6 h post-injury	76,912	72,968	971	2066.67	4.54	856
ME3.2				76,203	71,150	1081	1998.34	4.95	975
ME3.3				77,008	72,957	969	1830.47	4.02	863
ME4.1	Group ME4	oral	12 h post-injury	77,558	73,279	1174	1972.13	5.56	1047
ME4.2				74,410	69,148	1468	2464.91	6.77	1353
ME4.3				74,541	61,390	2806	3991.49	8.79	2762
ME5.1	Group ME5	oral	24 h post-injury	75,928	71,806	985	1931.79	4.61	885
ME5.2				74,611	69,202	1394	2224.90	6.53	1300
ME5.3				74,028	67,273	1459	2480.35	6.51	1376
ME6.1	Group ME6	oral	2 d post-injury	74,664	68,074	1229	2111.26	6.60	1142
ME6.2				75,143	70,151	1405	2415.12	6.57	1287
ME6.3				75,263	70,114	1465	2383.18	5.98	1353
ME7.1	Group ME7	oral	3 d post-injury	76,769	72,196	1344	2391.90	6.05	1220
ME7.2				76,083	66,074	2417	3860.69	6.20	2298
ME7.3				75,138	70,342	1340	2233.28	6.07	1235
ME8.1	Group ME8	oral	5 d post-injury	73,677	64,813	1646	2536.52	7.65	1586
ME8.2				73,510	67,294	1480	2269.39	6.76	1399
ME8.3				75,149	69,671	1307	2133.34	6.04	1211
ME9.1	Group ME9	oral	7 d post-injury	76,487	71,464	1041	1980.39	5.41	934
ME9.2				77,256	73,332	1412	2236.65	6.37	1280
ME9.3				76,074	72,053	1250	2131.06	5.38	1131
ME10.1	Group ME10	oral	10 d post-injury	74,698	64,240	2438	3771.06	7.41	2351
ME10.2				75,988	71,686	1417	2241.45	6.21	1230
ME10.3				77,097	72,449	1119	1926.33	4.38	1012
ME11.1	Group ME11	oral	14 d post-injury	73,656	68,661	1048	2039.08	4.96	958
ME11.2				77,171	71,986	1224	1992.04	5.56	1115
ME11.3				74,442	69,983	1351	2350.88	6.09	1243
FC1.1	Group FC1	fecal	Sham (0 h)	74,549	63,224	4644	6350.08	8.17	4526
FC1.2				78,141	69,901	4212	5464.17	8.02	3962
FC13				78,353	71,439	3795	4908.06	7.73	3545
FE1.1	Group FE1	fecal	0 h post-injury	72,912	62,697	4252	5677.05	8.04	4163
FE1.2				78,371	73,562	4248	5714.62	7.13	3893
FE1.3				75,060	67,562	3683	4874.46	7.75	3510
FE2.1	Group FE2	fecal	2 h post-injury	71,387	59,683	4025	5746.62	7.67	4009
FE2.2				76,979	71,123	3315	4509.30	7.16	3087
FE2.3				71,929	66,019	4046	5535.51	7.97	3873
FE3.1	Group FE3	fecal	6 h post-injury	76,357	69,373	4201	5769.69	7.50	3937
FE3.2				71,151	64,398	4662	6083.80	8.61	4527
FE3.3				76,482	71,302	4322	5782.13	7.58	4025
FE4.1	Group FE4	fecal	12 h post-injury	77,589	70,057	4805	6143.82	8.29	4525
FE4.2				75,067	68,011	5191	6714.32	8.97	4932
FE4.3				74,220	64,371	5193	7173.78	8.75	5021
FE5.1	Group FE5	fecal	24 h post-injury	75,389	69,399	4327	5750.95	7.55	4070
FE5.2				78,288	70,851	5166	6721.96	8.91	4832
FE5.3				76,998	72,103	4023	5302.52	6.92	3733
FE6.1	Group FE6	fecal	2 d post-injury	75,178	68,839	3498	4916.26	6.86	3285
FE6.2				75,699	68,243	4924	6399.17	8.72	4667
FE6.3				74,516	70,555	3178	4486.95	5.69	2948
FE7.1	Group FE7	fecal	3 d post-injury	78,591	72,364	3501	4790.55	6.66	3216
FE7.2				77,339	66,454	5600	7714.87	9.16	5342
FE7.3				76,786	71,029	3803	5228.15	6.62	3527
FE8.1	Group FE8	fecal	5 d post-injury	76,586	69,471	4473	5927.13	8.25	4206
FE8.2				77,399	70,549	4892	6248.60	8.84	4602
FE8.3				75,925	69,016	4343	5654.33	7.81	4094
FE9.1	Group FE9	fecal	7 d post-injury	77,773	69,274	4429	5948.41	8.49	4175
FE9.2				75,311	66,071	4749	6271.71	9.00	4559
FE9.3				75,555	67,513	4811	6270.56	8.99	4586
FE10.1	Group FE10	fecal	10 d post-injury	73,875	63,280	5193	7001.54	9.00	5059
FE10.2				78,491	68,669	4813	6325.99	8.88	4550
FE10.3				78,304	70,052	4902	6306.22	8.83	4607
FE11.1	Group FE11	fecal	14 d post-injury	77,742	68,515	4508	5955.91	8.09	4252
FE11.2				75,851	68,780	4427	5705.18	8.23	4183
FE11.3				77,711	67,696	5252	6594.63	9.21	5017

## Data Availability

The original data presented in this study was publicly available on NCBI. The all-raw sequences were stored in the sequence read file (accession number SRR18961860-SRR18961931).

## Supplementary Materials

The following supporting information can be downloaded at: https://www.mdpi.com/article/10.3390/microorganisms11061452/s1, Table S1: The details of Modified Neurological Severity Score Points (mNSS). One point is awarded for inability to perform the tasks for lack of a tested reflex: 13–18, severe injury; 7–12, moderate injury; 1–6, mild injury.
