# Supplementary material for: Temporal Changes of the Oral and Fecal Microbiota after Mild Traumatic Brain Injury in Rats by 16S rRNA Sequencing
Source: Microorganisms. 2023 May 31;11(6):1452. doi: 10.3390/microorganisms11061452 (PMC10301108; doi:10.3390/microorganisms11061452)
Supplement: Supplementary file 1 [file microorganisms-11-01452-s001.zip › microorganisms-2390251-supplementary.pdf]

Table S1. Modified Neurological Severity Score Points	
Motor tests	
Raising rat by tail	3
Flexion of forelimb	1
Flexion of hindlimb	1
Head moved >10º to vertical axis within 30s	1
Placing rat on floor (normal=0; maximum=3)	3
Normal walk	0
Inability to walk straight	1
Circling toward paretic side	2
Falls down to paretic side	3
Sensory tests	2
Placing test (visual and tactile test)	1
Proprioceptive test deep sensation, pushing paw against table edge to stimulate limb muscles)	1
Beam balance tests (normal=0; maximum=6)	6
Balance with steady posture	0
Grasps side of beam	1
Huge beam and 1 limb fall down from beam	2
Huge beam and 2 limb fall down from beam, or spins on beam (>60s)	3
Attempts to balance on beam but falls off (>40s)	4
Attempts to balance on beam but falls off (>20s)	5
Falls off; no attempt to balance or hang on to beam (<20s)	6
Reflex absence and abnormal movements	4
Pinna reflex (head shake when auditory meatus is touched)	1
Corneal reflex (eye blink when cornea is lightly touched with cotton)	1
Startle reflex (motor response to a brief noise from snapping a clipboard paper)	1
Seizures, myoclonus, myodystonia	1
Maximum points	18

Table S1. The details of Modified Neurological Severity Score Points (mNSS). One point is awarded for inability to perform the tasks of for lack of a tested reflex: 13-18, severe injury; 7-12 moderate injury; 1-6, mild injury.
